# Supplementary material for: Stage-specific expression patterns and co-targeting relationships among miRNAs in the developing mouse cerebral cortex
Source: Commun Biol. 2024 Oct 22;7:1366. doi: 10.1038/s42003-024-07092-7 (PMC11493953; doi:10.1038/s42003-024-07092-7)
Supplement: Supplementary file 3 — Description of Additional Supplementary File [file 42003_2024_7092_MOESM3_ESM.pdf]

Description Of Additional Supplementary File

File name: Supplementary Data 1
Description: Differentially expressed miRNAs between E14, E17 and P0 cortical samples as well as NPCs versus neurons.

File name: Supplementary Data 2
Description: Module assignment of miRNAs in the WCGNA analysis.

File name: Supplementary Data 3
Description: GO terms of miRNA targets of the black and green modules from the WCGNA analysis.

File name: Supplementary Data 4
Description: Significant co-targeting relationships between miRNAs/miRNA families.

File name: Supplementary Data 5
Description: Individual values underlying figures.
