# Supplementary material for: Stage-specific expression patterns and co-targeting relationships among miRNAs in the developing mouse cerebral cortex
Source: Commun Biol. 2024 Oct 22;7:1366. doi: 10.1038/s42003-024-07092-7 (PMC11493953; doi:10.1038/s42003-024-07092-7)
Supplement: Supplementary file 9 — Reporting Summary [file 42003_2024_7092_MOESM9_ESM.pdf]

Corresponding author(s): Susanne Gerber and Jennifer Winter

Last updated by author(s): Oct 10, 2024

Reporting Summary

Nature Portfolio wishes to improve the reproducibility of the work that we publish. This form provides structure for consistency and transparency in reporting. For further information on Nature Portfolio policies, see our Editorial Policies and the Editorial Policy Checklist.

Statistics

For all statistical analyses, confirm that the following items are present in the figure legend, table legend, main text, or Methods section.

n/a	Confirmed	
☐	☒	The exact sample size (*n*) for each experimental group/condition, given as a discrete number and unit of measurement
☐	☒	A statement on whether measurements were taken from distinct samples or whether the same sample was measured repeatedly
☐	☒	The statistical test(s) used AND whether they are one- or two-sided<br>*Only common tests should be described solely by name; describe more complex techniques in the Methods section.*
☐	☒	A description of all covariates tested
☐	☒	A description of any assumptions or corrections, such as tests of normality and adjustment for multiple comparisons
☐	☒	A full description of the statistical parameters including central tendency (e.g. means) or other basic estimates (e.g. regression coefficient) AND variation (e.g. standard deviation) or associated estimates of uncertainty (e.g. confidence intervals)
☐	☒	For null hypothesis testing, the test statistic (e.g. *F*, *t*, *r*) with confidence intervals, effect sizes, degrees of freedom and *P* value noted<br>*Give P values as exact values whenever suitable.*
☒	☐	For Bayesian analysis, information on the choice of priors and Markov chain Monte Carlo settings
☒	☐	For hierarchical and complex designs, identification of the appropriate level for tests and full reporting of outcomes
☐	☒	Estimates of effect sizes (e.g. Cohen's *d*, Pearson's *r*), indicating how they were calculated

Our web collection on statistics for biologists contains articles on many of the points above.

Software and code

Policy information about availability of computer code

Data collection	R version 4.2.2, Microsoft Excel
Data analysis	miRDeep v2.0.1.2 for preprocessing of small RNA-seq, BBDuk v39.01 for adapter trimming, STAR v2.7.10b for alignment, SubRead v2.0.6 for feature counting, R v4.2.2 for statistical data analysis and visualization including the R packages DESEq2 v1.40.1 for differential expression analysis, edgeR v3.30.3 for normalization of RNA-seq data, clusterProfiler v4.6.2 for functional annotation analysis, bnlearn v4.8.3 for network reconstruction, KDA v0.2.2 for hub gene analysis and custom code for co-targeting prediction.

For manuscripts utilizing custom algorithms or software that are central to the research but not yet described in published literature, software must be made available to editors and reviewers. We strongly encourage code deposition in a community repository (e.g. GitHub). See the Nature Portfolio guidelines for submitting code & software for further information.

Data

Policy information about availability of data

All manuscripts must include a data availability statement. This statement should provide the following information, where applicable:

- Accession codes, unique identifiers, or web links for publicly available datasets
- A description of any restrictions on data availability
- For clinical datasets or third party data, please ensure that the statement adheres to our policy

The raw RNA-seq data were uploaded to the Sequence Read Archive (SRA) data base under the accession number PRJNA1018560.

The following supplementary tables are available online under 10.5281/zenodo.1016183:
Supplementary_table_1.xlsx: Differentially expressed miRNAs between E14, E17 and P0 cortical samples as well as NPCs versus neurons
Supplementary_table_2.xlsx: Module assignment of miRNAs in the WCGNA analysis.
Supplementary_table_3.xlsx: GO terms of miRNA targets of the black and green modules from the WCGNA analysis.
Supplementary_table_4.xlsx: Significant co-targeting relationships between miRNAs/miRNA families.

Research involving human participants, their data, or biological material

Policy information about studies with human participants or human data. See also policy information about sex, gender (identity/presentation), and sexual orientation and race, ethnicity and racism.

Reporting on sex and gender	N/A
Reporting on race, ethnicity, or other socially relevant groupings	N/A
Population characteristics	N/A
Recruitment	N/A
Ethics oversight	N/A

Note that full information on the approval of the study protocol must also be provided in the manuscript.

Field-specific reporting

Please select the one below that is the best fit for your research. If you are not sure, read the appropriate sections before making your selection.

☒ Life sciences ☐ Behavioural & social sciences ☐ Ecological, evolutionary & environmental sciences

For a reference copy of the document with all sections, see nature.com/documents/nr-reporting-summary-flat.pdf

Life sciences study design

All studies must disclose on these points even when the disclosure is negative.

Sample size	No formal sample size calculation was performed. For sequencing of cerebral cortex samples, 6 biological replicates per condition were used to account for the increased variability of in vivo samples. For sequencing of NPC and neurons, three replicates per group were used due to the lower between sample variability of in vitro samples.
Data exclusions	2 samples from the sequencing of the embryonic cerebral cortex were excluded prior to the analysis due to low number of sequencing reads.
Replication	Expression patterns of miRNAs analysed in the cerebral cortex of mice at developmental stages E14, E17 and P0 were replicated in an independent sequencing run in neural progenitor cells differentiated into neurons. For selected miRNAs, expression patterns in E14, E17, P0 samples as well as in progenitor cells and neurons were also confirmed using qPCR.
Randomization	N/A
Blinding	Investigators were not blinded during data collection and analysis.

Reporting for specific materials, systems and methods

We require information from authors about some types of materials, experimental systems and methods used in many studies. Here, indicate whether each material, system or method listed is relevant to your study. If you are not sure if a list item applies to your research, read the appropriate section before selecting a response.

Materials & experimental systems

n/a	Involved in the study
☒	☐ Antibodies
☒	☐ Eukaryotic cell lines
☒	☐ Palaeontology and archaeology
☐	☒ Animals and other organisms
☒	☐ Clinical data
☒	☐ Dual use research of concern
☒	☐ Plants

Methods

n/a	Involved in the study
☒	☐ ChIP-seq
☒	☐ Flow cytometry
☒	☐ MRI-based neuroimaging

Animals and other research organisms

Policy information about studies involving animals; ARRIVE guidelines recommended for reporting animal research, and Sex and Gender in Research

Laboratory animals	NMRI mouse embryos were used for small RNA sequencing at E14, E17 and P0
Wild animals	N/A
Reporting on sex	Female and male mouse embryos were used for small RNA sequencing of the embryonic cerebral cortex. Principal component analysis of the expression data did not reveal a sex effect, therefore both sexes were pooled together for the subsequent analyses.
Field-collected samples	N/A
Ethics oversight	Ethical review and approval were not required for the animal study because the study did not include any animal experiments requiring approval. To carry out this study, mice were killed for organ removal. In Germany, this procedure is notifiable but does not require approval by an ethics committee.

Note that full information on the approval of the study protocol must also be provided in the manuscript.

Plants

Seed stocks	N/A
Novel plant genotypes	N/A
Authentication	N/A
